# Lower-extremity joint kinematics and muscle activations during semi-reclined cycling at different workloads in healthy individuals

**DOI:** 10.1186/1743-0003-11-146

**Published:** 2014-10-17

**Authors:** Kamyar Momeni, Pouran D Faghri, Martinus Evans

**Affiliations:** Biomedical Engineering Department, University of Connecticut, Storrs, Connecticut USA; Department of Allied Health Sciences, University of Connecticut, Storrs, Connecticut USA

## Abstract

**Background:**

A better understanding of lower-extremity muscles’ activation patterns and joint kinematics during different workloads could help rehabilitation professionals with prescribing more effective exercise regimen for elderly and those with compromised muscles. We examined the relative contribution, as well as activation and co-activation patterns, of lower-extremity muscles during semi-reclined cycling at different workloads during a constant cadence.

**Methods:**

Fifteen healthy novice cyclists participated at three 90-second cycling trials with randomly assigned workloads of 0, 50, and 100 W, at a constant cadence of 60 rpm. During all trials, electromyograms were recorded from four lower-extremity muscles: rectus femoris (RF), biceps femoris (BF), tibialis anterior (TA), and gastrocnemius medialis (GT). Joint kinematics were also recorded and synchronized with the EMG data. Muscle burst onset, offset, duration of activity, peak magnitude, and peak timing, as well as mean joint angles and mean ranges of motion were extracted from the recorded data and compared across workloads.

**Results:**

As workload increased, BF and TA displayed earlier activations and delayed deactivations in each cycle that resulted in a significantly (p < 0.05) longer duration of activity at higher workloads. RF showed a significantly longer duration of activity between 0 and 50 W as well as 0 and 100 W (p < 0.05); however, the activity duration of GT was not appeared to be affected significantly by workload. EMG peak-magnitude of RF, BF, and TA changed significantly (p < 0.05) as workload increased, but no changes were observed in the EMG peak-timing across workloads. Durations of co-activation in the RF-BF pair as well as the RF-TA pair increased significantly with workload, while the RF-TA and TA-GT pairs were only significantly different (p < 0.05) between the 0 and 100 W workload levels. Increased workload did not lead to any significant changes in the joint kinematics.

**Conclusions:**

Muscles’ activity patterns as well as co-activation patterns are significantly affected by changes in cycling workloads in healthy individuals. These variations should be considered during cycling, especially in the elderly and those with compromised musculoskeletal systems. Future research should evaluate such changes specific to these populations.

**Electronic supplementary material:**

The online version of this article (doi:10.1186/1743-0003-11-146) contains supplementary material, which is available to authorized users.

## Background

Cycling intervention could be an ideal exercise for those with compromised musculoskeletal system such as the elderly, following rehabilitation or prolonged bed rest [1–5]. The cycling exercise potentially minimizes the stress on the joints when compared to weight bearing exercises and could have beneficial effects on mobility and functional abilities in performing daily activities [1, 6]. It could further improve muscle strength, power, and joints’ ranges of motion, while providing significant cardiovascular benefits [1]. Compared to upright cycling, differences in physical characteristics of semi-reclined bicycles, such as larger seat, back support, lower profile, and side handgrips [3], make semi-reclined cycling more appealing to patients during rehabilitation. Accordingly, many factors need to be considered when utilizing cycling as an exercise or rehabilitation intervention. For instance, studies have shown that the positioning such as seat height, crank’s arm length, and foot position in which an individual cycles could affect the biomechanical efficacy of the cycling [7, 8]. Recumbent cycling has also been recommended as the better choice due to less stress on the joints and muscles and its more comfortable platform [3, 6].

Both cycling cadence and workload have been shown to affect joint moments, muscle activation patterns, and overall energy expenditure. It has been reported in upright cycling that as joint moments (hip, knee, and ankle) increase, pedal forces start to decrease. Redfield and Hull [9] reported that the hip moment was the most significantly affected due to its involvement in acceleration and deceleration. This could be an indication of higher demand on the hip joint during higher cadences. In rehabilitation programs or among the elderly, high cycling cadences lowers the individual’s efficiency and may cause injury to already compromised or frail hip joints [10, 11]. On the other hand, increases in workload cause an increase in both knee and ankle moments [3] and consequently, may reduce the pressure on the hip joint at lower cadences.

In a study involving recreational cyclists, increasing resistance during constant cadence cycling caused an increase in peak pedal force, indicating more strength training on the muscle. However, increasing cadence while keeping power output constant led to a decrease in peak pedal force. While there are many studies on the effects of workload and cadence during upright cycling, there is a paucity of research evaluating semi-reclined cycling. For instance, Gregor et al. [3] compared generalized muscle moments (GMM) at the hip, knee, and ankle across different workloads during semi-reclined cycling and stated that the magnitude of GMM values increased by workload. Furthermore, they compared their findings to available GMM data on upright cycling and reported lower GMM magnitudes during semi-reclined cycling. More research is needed to investigate kinematics and muscle activity patterns during semi-reclined cycling in order to improve rider’s performance and ergometer’s design.

The purpose of the present study was to evaluate the relative contribution of upper and lower leg muscles as well as muscles’ functioning (activation and synchronization patterns) during semi-reclined cycling at different workloads during constant cadence using electromyography (EMG) and kinematics analysis. The findings may help rehabilitation professionals to design better exercise regimen for older adults and to develop more effective rehabilitation programs for those in need.

## Methods

### Participants

Fifteen healthy, male, novice cyclists participated in this study. Their age, height, and weight were 22 ± 2 yr, 1.79 ± 0.08 m, and 74 ± 7 kg, respectively. Participants who had history of bone and/or joint problems or those who responded positively to any of the questions on the Physical Activity Readiness Questionnaire (PAR-Q) were excluded [12]. PAR-Q is a self-administered questionnaire used as a preliminary to fitness testing. The test is recommended for individuals between the ages of 15 and 69 years; it determines the safety or potential risk for the person, based on certain health history questions. All participants signed a consent form approved by the university Institutional Review Board (IRB). The study was conducted in the Functional Performance Laboratory, in a university setting.

### Instrumentation

#### Semi-reclined stationary ergometer

**Figure 1:**
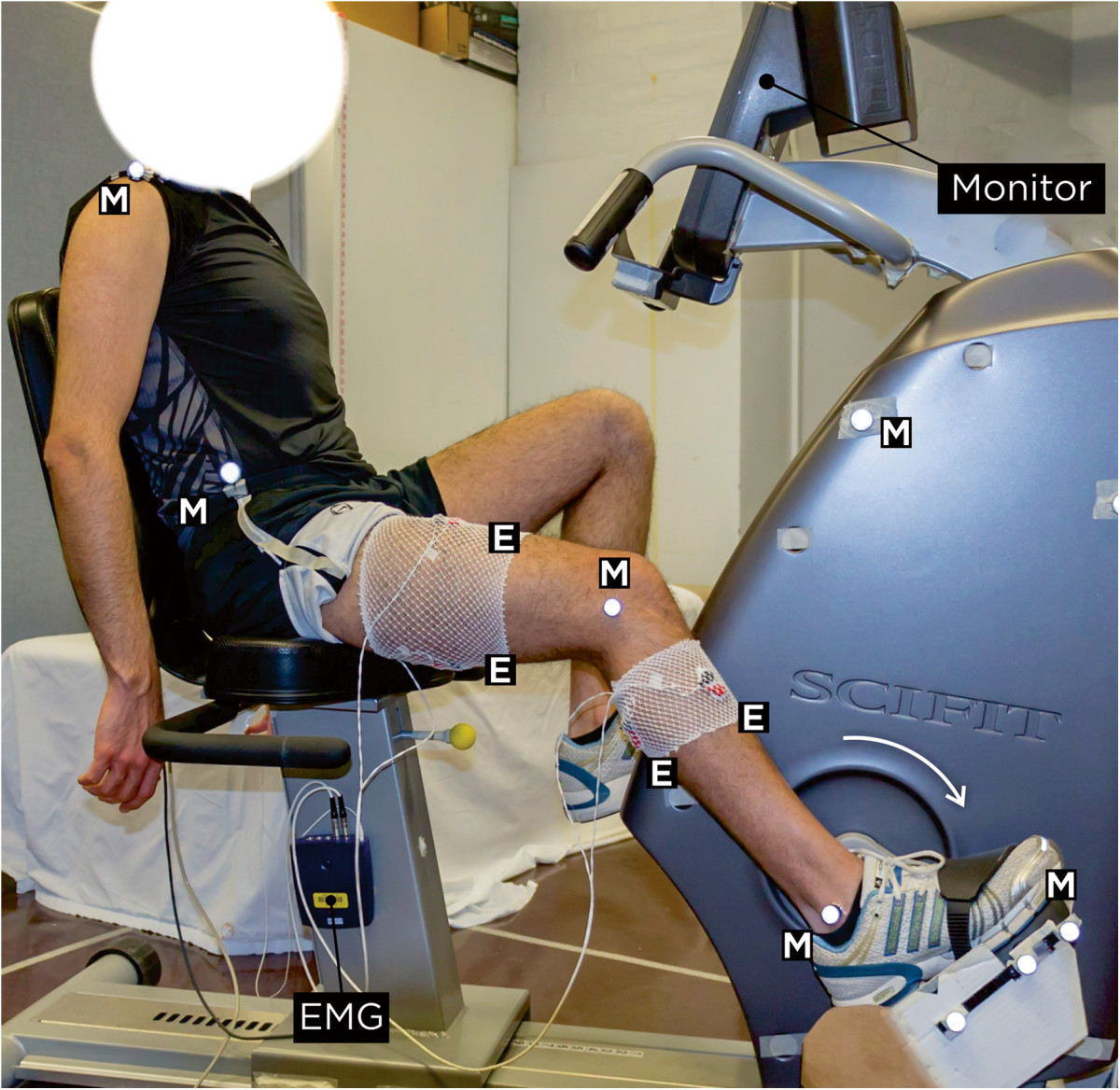
**Experimental setup.** E: four bipolar pre-gelled Ag-AgCl surface EMG electrodes with a 2 cm inter-electrode distance. M: retroreflective markers.

A standard stationary semi-reclined ergometer (SciFit ISO 7000R, Tulsa, OK) was used throughout the study [5]. The semi-reclined position implies a slightly inclined torso with pedals near seat height. The stationary bike was equipped with a speedometer and foot straps, utilized to ensure the fixed position of the foot on the pedal surface while enforced no limitations on the movement of the ankle. Although the bike enforced no other movement restrictions, participants were asked to remain seated during the cycling trials and keep their arms hanging on the sides of the bike (Figure 1).

#### Motion capture system

A Vicon motion capture system (VICON Motion Systems Ltd., United States) was used to record body movements as well as pedal and crank positions continuously. Retroreflective markers were placed on shoulder (acromioclavicular joint), hip (greater trochanter), knee (lateral epicondyle of femur), ankle (lateral malleolus), lateral aspect of fifth metatarsal head (on sneaker approximate to the landmark), and calcaneus. An additional four markers were placed on the pedal spindle, crank axle, and two reference points on the body of the bike.

#### Surface electromyography

A 10-channel physiological monitoring system (Nexus-10, MindMedia B.V., Netherlands) was utilized to record surface EMG signals of four lower limb muscles: Rectus Femoris (RF), Biceps Femoris (BF), Tibialis Anterior (TA), and Gastrocnemius Medialis (GT). Electrode locations were determined by following the recommendations of the SENIAM project (Surface Electromyography for the Non-Invasive Assessment of Muscles). Bipolar pre-gelled silver/silver-chloride (Ag-AgCl) surface electrodes, with a 2-cm inter-electrode distance, were used (Noraxon USA Inc., Scottsdale, AZ).

### Experimental protocol

Participants completed a questionnaire, which recorded their demographics as well as physical activity behavior, using the International Physical Activity Questionnaire (IPAQ) [13]. IPAQ is an instrument for monitoring and quantifying an individual’s level of physical activity. It is recommended for use among adults between the ages of 18 to 65. Height and weight were measured and body mass index (BMI) was calculated [14]. Resting heart rate and blood pressure were also measured following a 10-minute rest period on a chair. All heart rate and blood pressure measurements were performed by a trained researcher, using calibrated equipment.

### Subject preparation

Conforming to the recommendations of the SENIAM, electrode sites were identified, shaved, lightly abraded, and cleaned using alcohol swabs to reduce skin impedance. One bipolar surface electrode (Myotronics-Noromed Inc., Kent, WA) was placed on the midpoint of the contracted muscle belly, parallel to the muscle fibers of each muscle. A common reference electrode was positioned on a bony site, at the distal end of the left ulna. Ball-shaped retroreflective markers were placed over the skin, using double-sided tapes, on the specified anatomical landmarks. To avoid possible artifacts, all the markers, electrodes, and wires were fixed on the skin by using adhesive tapes. Preparations completed by adjusting the seat on the bike such that the minimal knee flexion angle of the right leg was measured at 40°, while the pedal was at 110° clockwise from vertical. Seat adjustment was performed by moving the bicycle’s seat horizontally towards or away from the pedal. Prior to testing, participants pedaled at a self-selected cadence with no resistance for 5 minutes. This process helped participants to experience and feel comfortable with the bike throughout the experimental protocol.

### Experimental protocol

Each participant performed three 90-second trials at three workloads of 0, 50, and 100 Watts (W), and was encouraged to maintain a constant cadence of 60 ± 3 rotations-per-minute (rpm) throughout the testing period. A monitor in front of the bike, visible by both the rider and the researcher, continuously displayed the cycling speed in rpm. This was used to provide feedback to the participant to continue maintaining the cadence at 60 rpm throughout the trial. Throughout the experiment, all participants remained within the ±3 rpm acceptable cadence. Resistance levels were adjusted accordingly to achieve the target power output in each trial (i.e., 0, 50, 100 W). The testing order of the trials was randomized for each participant to minimize potential order effects and 5-minute resting periods was allowed between each trial to avoid possible fatigue effects. Heart rate and blood pressure were monitored during all trials. Heart rate was monitored continuously throughout the experiment, using a wireless Polar T34 chest-belt heart rate transmitter. The researcher measured blood pressure before and after each trial, using an Omron HEM-712C automatic blood pressure monitor (Omron Healthcare Inc., Kyoto, Japan). Each trial consisted of a 2-minute warm up, a 90-second constant cadence cycling at a given workload, and a 2-minute cool down.

### Data collection

Participants cycled for 90 seconds during each trail. After 30 seconds, we randomly selected a 30-second window for collecting data on approximately 30 revolutions while pedaling. Raw EMG data were recorded at 2048 Hz, while kinematic data were collected at 128 frames-per-second (fps). Recorded data were synchronized by using a custom-written Labview (National Instruments Corp., Austin, TX) program and imported into Matlab (MathWorks Inc., Natick, MA) for further data processing and statistical analyses. EMG data were down-sampled to 360 Hz, while the kinematic data were resampled by piece-wise linear interpolation in order to make it synchronous to the relative down-sampled EMG signal.

### Data processing

#### Kinematics data

**Figure 2:**
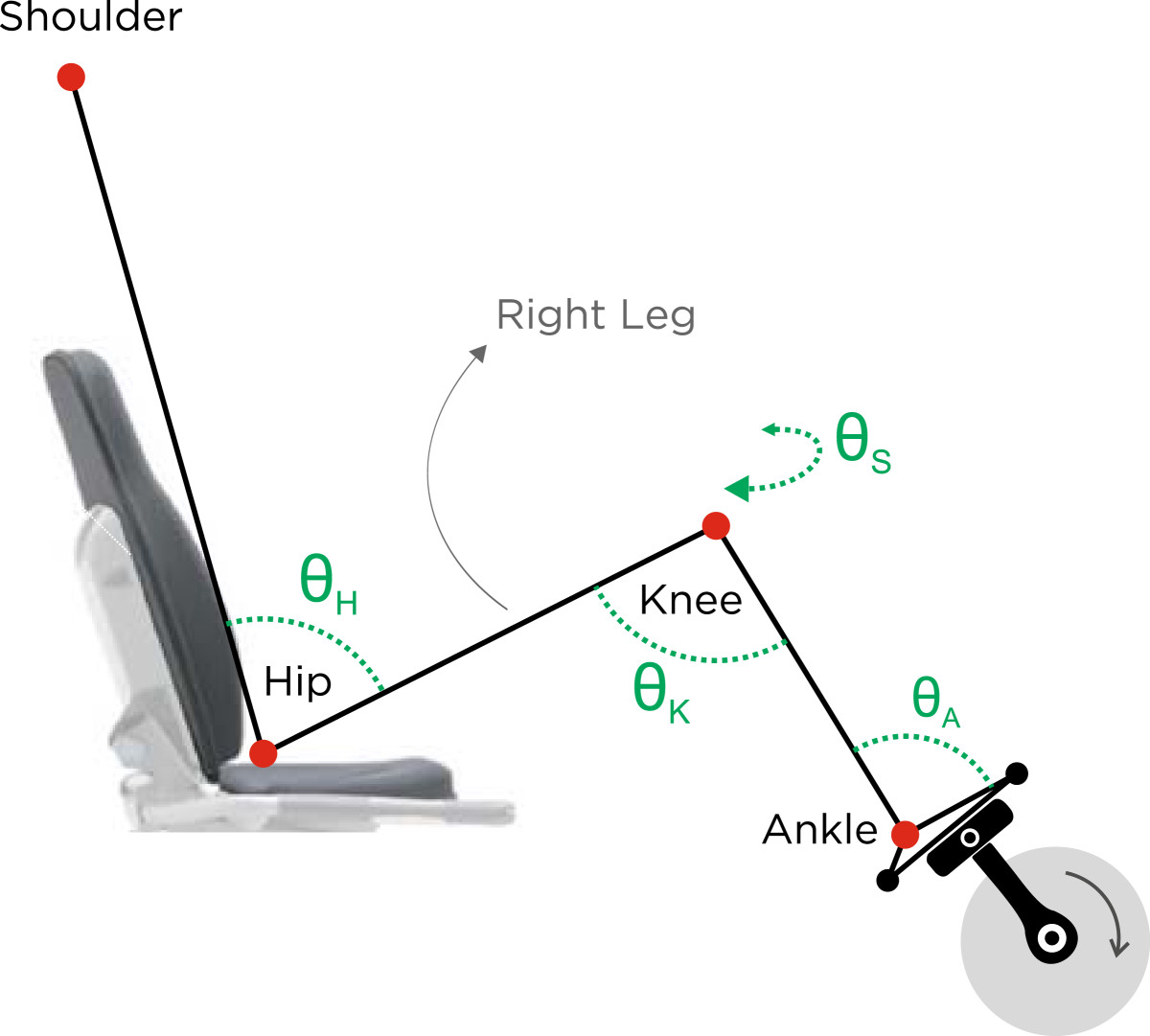
**Schematic illustration of angles and body position.** Angles are defined on the sagittal plane: hip (Ө_H_), knee (Ө_K_), and ankle (Ө_A_) angles, and on the transverse plane: knee splay (Ө_S_).

**Figure 3:**
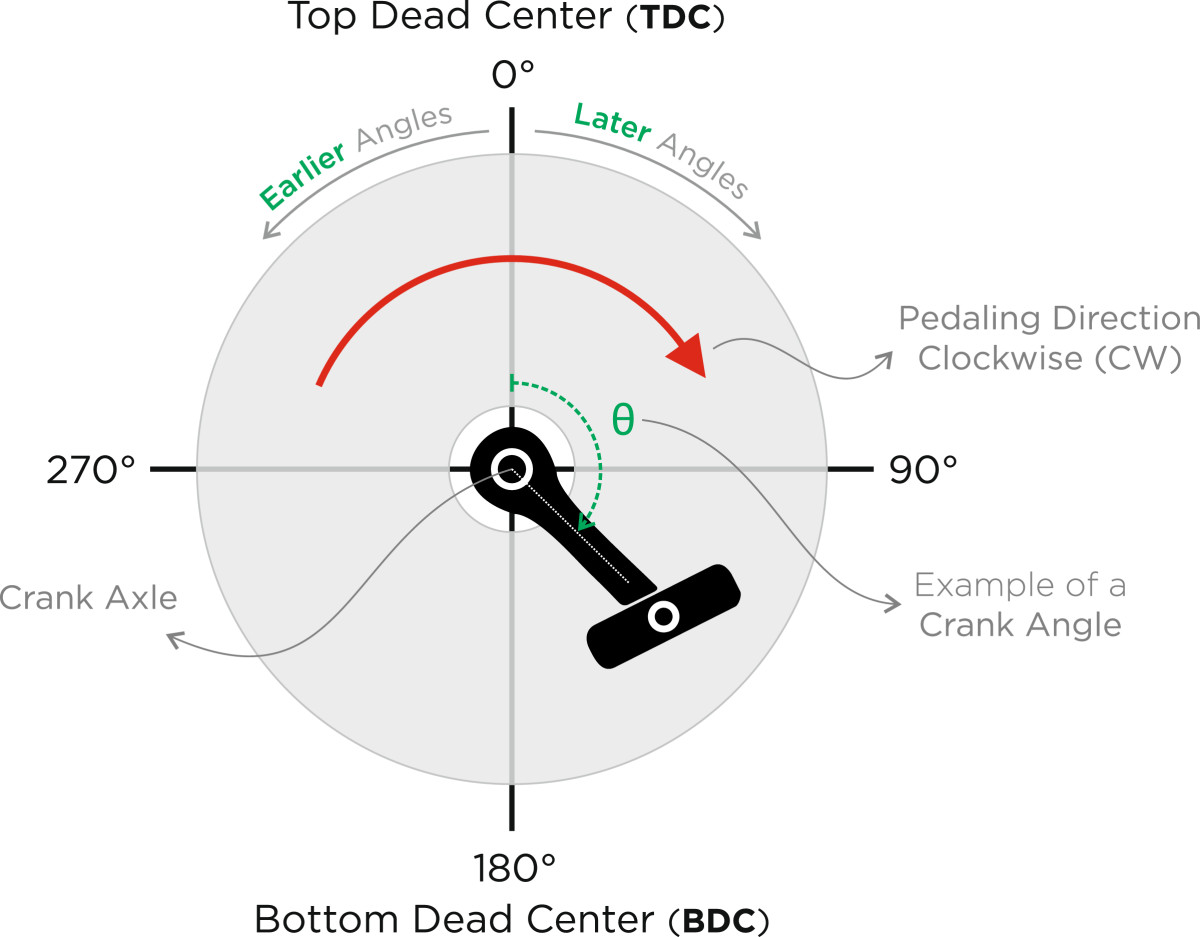
**Schematic illustration of the right foot’s pedal.** Pedaling direction is assumed clockwise (CW) in this illustration. A full cycle of the pedal starts from 0°, continues CW, and ends at 360° (i.e., 0°).

Raw 3D coordinate data were filtered in Matlab® by using a low-pass, 4th-order, zero-lag Butterworth filter with a cut-off frequency of 10 Hz. Crank angles were calculated and used to identify the location of the pedal (Figure 2). For each participant, ensemble average of 20 consecutive full revolutions of the crank (0°-360°) was calculated at every workload (Figure 3). Hip (Ө_H_), knee (Ө_k_), and ankle (Ө_A_) angles were calculated on the sagittal plane. In the transverse plane, the thigh abduction/adduction angle (Ө_S_) was also calculated. Joint angles were utilized to investigate any possible changes in the kinematic patterns across different workloads. In order to achieve this goal, two dependent variables were considered for all calculated angles: Mean Joint Angles (MA) and Ranges of Motion (ROM). MA represents the average of joint angle values, while ROM is the subtraction of the minimum from the maximum angle. These measures quantified the amount of change in movement kinematics during cycling across different workloads [15, 16].

#### Electromyography data

Following the manufacturer’s recommendations, raw EMG signals were bandpass filtered from 20 to 500 Hz using a 5th-order Butterworth filter using the native software of the device, BioTrace + (MindMedia B.V., Netherlands). After preliminary filtering, data were transmitted to Matlab® for further processing. Signals were then full-wave rectified and smoothed by a low-pass, zero-lag, 4^th^-order Butterworth filter with a cut-off frequency of 5 Hz to form the linear envelope. The EMG ensemble average (EEA) [2] was calculated for 20 consecutive cycles for each workload and was scaled to a percentage of the identified maximum EMG amplitude for each muscle. The following parameters were then extracted from the resultant curves: EMG burst onset (EMG_on_), EMG burst offset (EMG_off_), duration of EMG activity (EMG_d_), EMG burst peak-timing (EMG_t_), and EMG burst peak magnitude (EMG_peak_). All variables were calculated in units of degrees (°) except for EMG_peak_ calculated in microvolts (μV). EMG burst onset and offset criteria were defined by a threshold value of 10% of the peak EMG magnitude across all workloads for each participant and were identified using Matlab. If necessary, visual analysis was performed and the threshold, in the Matlab code, was automatically increased to 15% or 20%, until the appropriate threshold was reached. Muscle was considered active while EMG signal reached the specified threshold and inactive while it dropped below this value. The duration, in degrees, between a muscle activation (i.e., onset) and its consecutive deactivation (i.e., offset) was referred to as the duration of activity (EMG_d_):

$$EMG_{d}\;=\;EMG_{\mathrm{off}}-EMG_{\mathrm{on}}$$

EMG_peak_ was the maximum EMG magnitude of each muscle’s EEA curve at each workload and EMG_t_ was the crank angle at which the EMG_peak_ occurred. Activation of agonist and antagonist muscles, known as co-activation, were examined for four muscle pairs (i.e., RF-BF, TA-GT, RF-TA, and RF-GT) by using the identified onset and offset points. For each muscle pairs, the start and end of co-activations, in terms of crank angles, were identified for every workload and the co-activation duration was calculated, in degrees, as the difference between the crank angle values of the start and the end of each co-activation.

### Statistical analysis

A one-way within-subjects analysis of variance (ANOVA) with repeated measures was performed to investigate the differences in the duration of EMG activity (EMG_d_), EMG burst peak magnitude (EMG_peak_), and duration of co-activation as well as cycling kinematics (i.e., MA and ROM), across the three workloads. Tukey’s HSD post-hoc analysis was utilized when necessary. Significance level was established at p-value < 0.05 for all analyses. For the remaining EMG variables (i.e., EMG_on_, EMG_off_, EMG_t_), circular statistics (i.e., directional statistics) [17, 18], was used to calculate descriptive statistics and investigate the differences between the three workloads. Said variables are temporal measures associated with an angle θ of the crank’s arm, which makes 0° and 360° identical angles. In this case, 180° cannot be reported as the mean of 5° and 355°. To avoid introducing such discontinuity to the data, circular statistics was used as the suitable method for analyzing these variables. Descriptive circular statistics, including the mean crank angle and circular standard deviation, were calculated by transforming Cartesian coordinates into polar coordinates.

## Results

**Figure 4:**
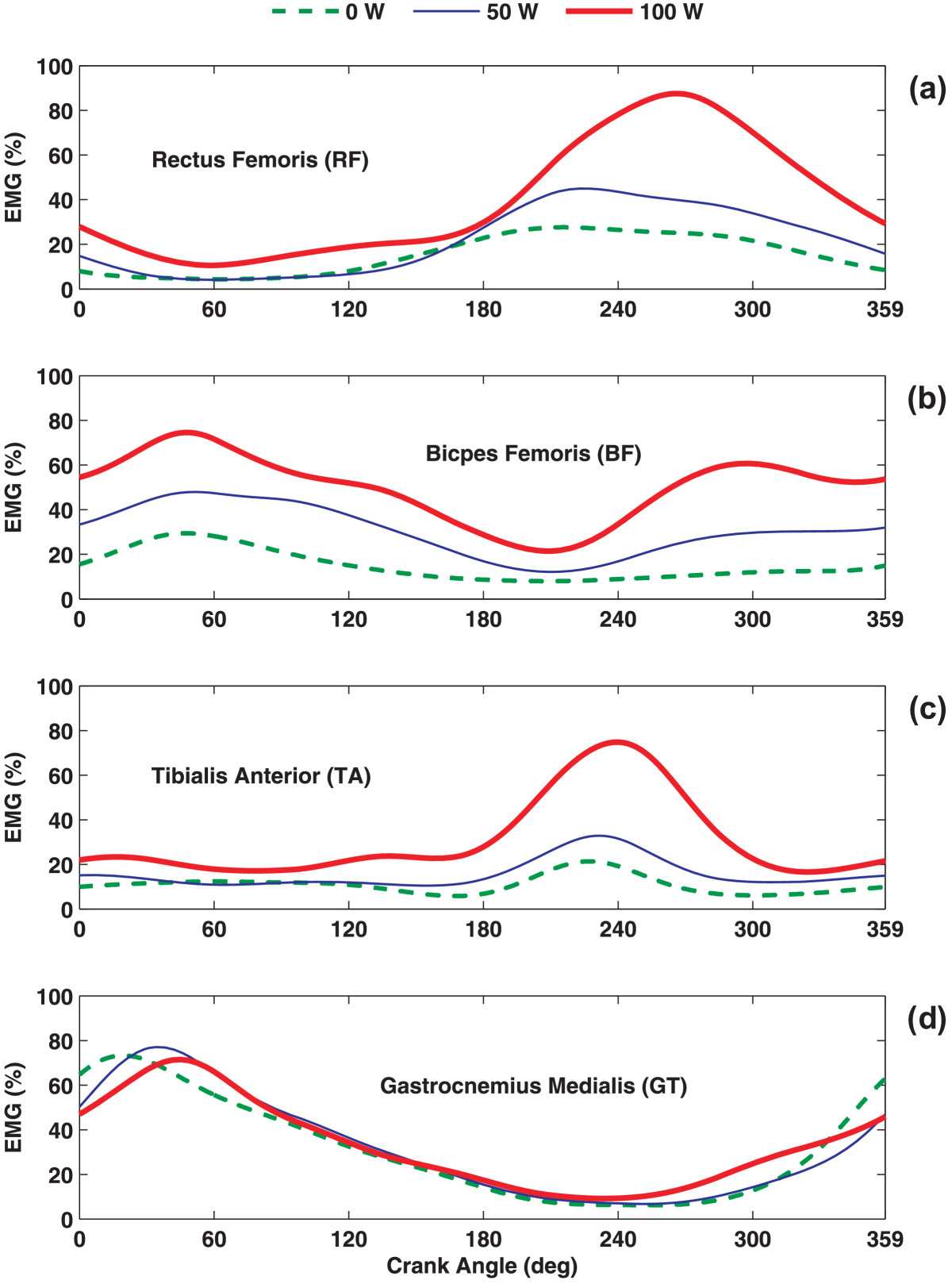
**EMG ensemble average (EEA) curves.** EEA curves of all participants’ EMG linear envelopes across three workload conditions for **(a)** RF, **(b)** BF, **(c)** TA, and **(d)** GT muscles. The crank angle represents TDC to its next TDC, which is 0° to 360°.

Demographic information of the participants is presented in Table 1. The analysis of the IPAQ responses revealed that on average, participants were considered moderately active with a median IPAQ score of 1522.50 MET (Metabolic Equivalent of Task) min/week [13].Participants’ muscle activity patterns for the three workloads (i.e., 0, 50, 100 W) represented with the EMG ensemble average (EEA) curves are illustrated in Figure 4.

**Table 1:** **Demographic information of participants**

Age	Height	Weight	BMI	IPAQ Score	IPAQ Level
(yr)	(m)	(kg)	(kg/m^2^)	(MET min/week)	Median
Mean ± SD	Mean ± SD	Mean ± SD	Mean ± SD	Median	
22.27 ± 1.83	1.79 ± 0.08	74.09 ± 6.99	23.12 ± 2.07	1522.50	Moderate

### Onset/offset

**Figure 5:**
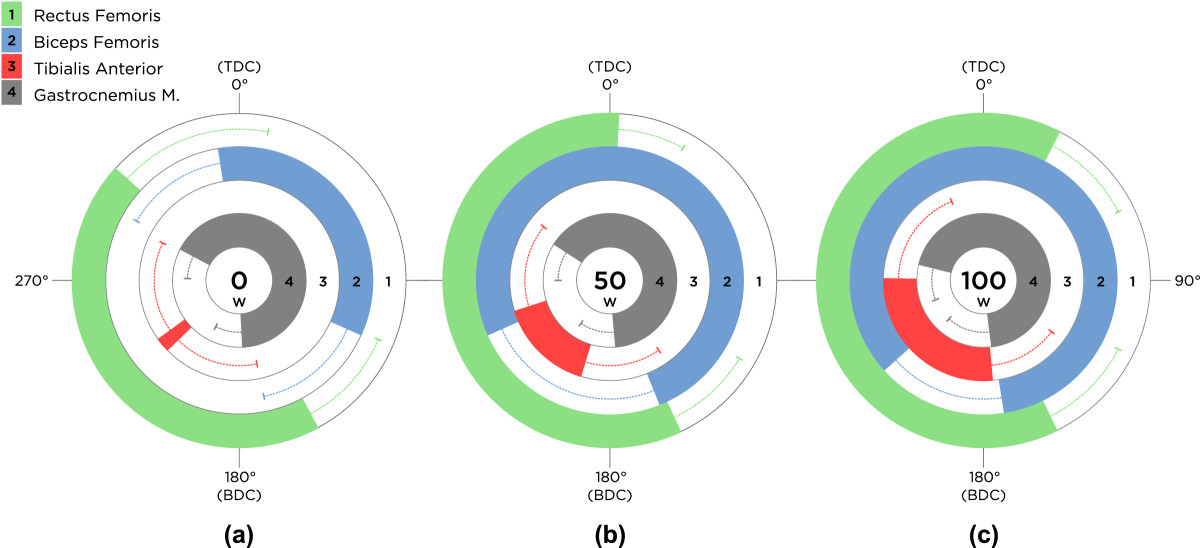
**Activity of lower limb muscles across workloads.** Mean EMG burst onset, offset, and duration of activity of all participants’ EMG linear envelopes of rectus femoris (RF), biceps femoris (BF), tibialis anterior (TA), and gastrocnemius medialis (GT) across the three workload levels of **(a)** 0 W, **(b)** 50 W, and **(c)** 100 W. Movement of the pedal is assumed clockwise (CW) in all conditions, starting from TDC. Error bars represent one standard deviation (SD) of the mean onset or offset.

As cycling workload increased, onset of BF and TA showed consistent activity shifts toward earlier angles of the pedal, which indicates relatively sooner activation in the pedaling cycle (Figure 5). On the contrary, RF and GT exhibited small and inconsistent changes in their onset across workloads. More specifically, the onset of the RF muscle was slightly delayed as workload increased from 0 to 50 W and from 0 to 100 W, while the onset of GT occurred marginally earlier in the cycle at 100 W, compared to 0 or 50 W (Figure 5). Evaluation of EMG burst offset indicated that RF, BF, and TA exhibited delayed deactivations (i.e., occur at a later crank angle, in the direction of cycling), as workload increased during cycling (Figure 5). In contrast, for GT, as workload increased, the offset angles appeared slightly earlier showing a shift towards sooner deactivations in relation to higher workloads during cycling.

### Duration

The duration of activity in both BF and TA showed significant increases (p < 0.05) as workload increased, while RF only displayed a significantly longer duration of activity between 0 W and the two higher workloads: 50 and 100 W (Figure 5). With increasing workload, duration of activity in RF, BF, and TA increased. GT showed a decrease in duration of activity as workload increased from 0 to 50 W; however, its duration of activity increased to its largest value at 100 W.

### EMG burst peak-magnitude

**Figure 6:**
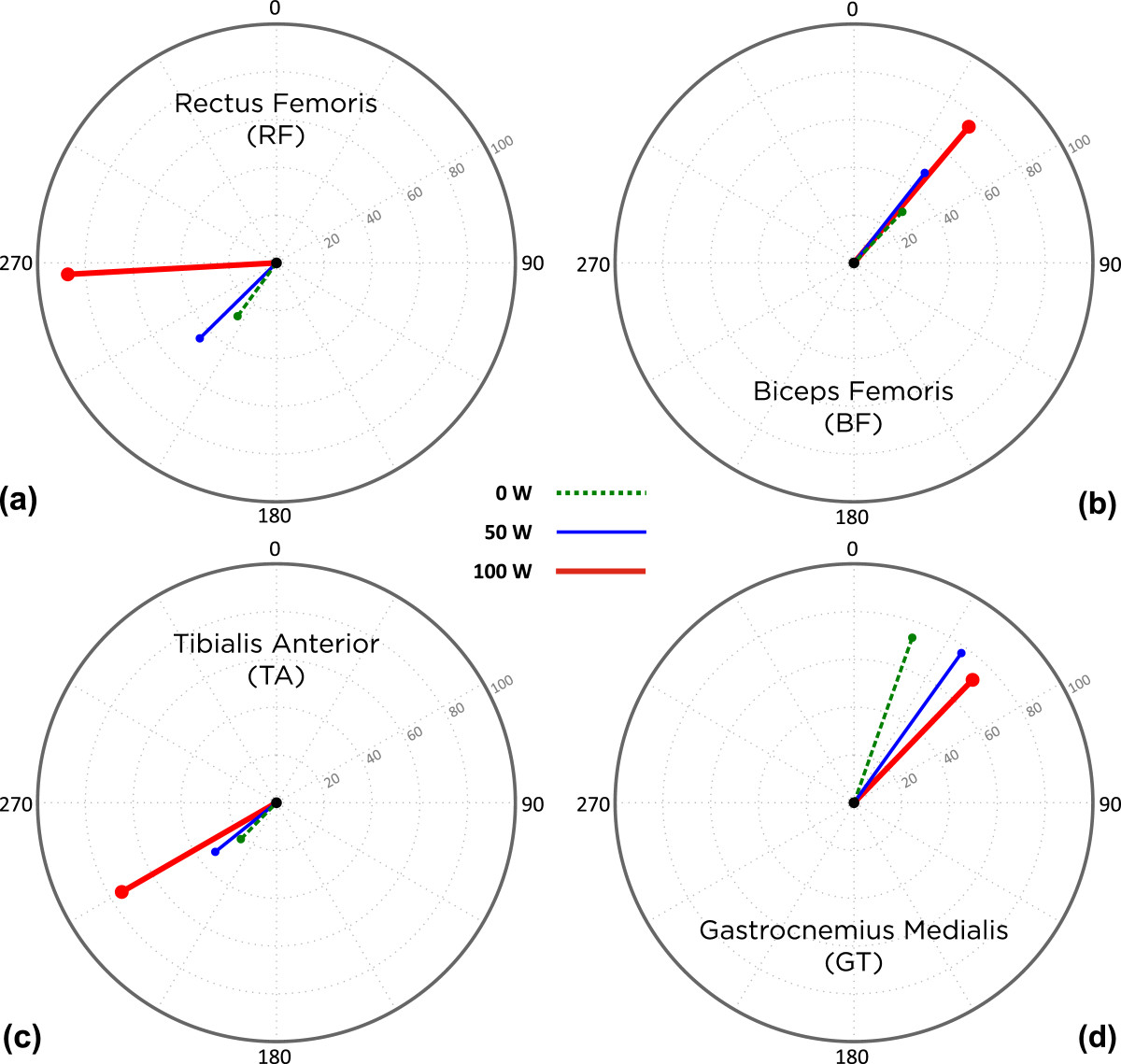
**Peak EMG magnitude and timing.** Mean peak EMG magnitude and timing, in terms of crank angles, of all participants across the three workloads for **(a)** RF, **(b)** BF, **(c)** TA, and **(d)** GT muscles. Length of each line represents the normalized mean peak EMG magnitude and points at its timing, where the peak had occurred during the crank’s rotation.

EMG burst peak magnitude values of each muscle were identified after all signals were normalized over the maximum EMG magnitude detected across all three workload conditions within each participant (Figure 6). RF and TA showed significant differences (*p* < 0.05) in EMG peak magnitude in response to an increase in workload from 0 to 100 W and 50 to 100 W (Table 2). BF demonstrated significant differences (*p* < 0.05) in EMG peak magnitude values between all three levels of workload. And yet, GT did not show any significant changes in EMG peak magnitude values across workloads.

**Table 2:** **Mean EMG burst peak magnitude of the normalized EMG signals (%)**

Workload (Watts) →	0	50	100
**RF**	23 (15) #	35 (19) ◊	66 (34) #,◊
**BF**	7 (4) *,#	13 (6) *,◊	22 (9) #,◊
**TA**	21 (24) #	30 (31) ◊	65 (37) #,◊
**GT**	59 (27)	65 (25)	63 (28)

Values are mean (±SD).

*: Statistical significance between workloads 0 and 50 W (p-value < 0.05).

#: Statistical significance between workloads 0 and 100 W (p-value < 0.05).

**◊**: Statistical significance between workloads 50 and 100 W (p-value < 0.05).

### EMG burst peak-timing

EMG peak-timing was identified as the angle of the crank where the EMG burst peak magnitude was observed (Figure 6). For RF and GT, the EMG peak magnitude occurred later in the cycling phase as workload increased; however, no consistent pattern was observed in the EMG peak-timing of BF and TA. None of the muscle groups showed statistically significant changes in the timing of the peak EMG magnitude while workload increased.

### Co-Activation duration

Throughout all workload levels, co-activations of four muscle pairs were evaluated: 1) RF-BF, 2) TA-GT, 3) RF-TA, and 4) RF-GT. As workload increased, the durations of co-activations, in terms of crank angles, were numerically increased in a consistent manner for every muscle pair. The RF-BF pair displayed a significant (*p* < 0.05) increase in co-activation across all workloads (Table 3). Similar to the RF-BF pair, RF-TA pair showed significant (*p* < 0.05) differences throughout the three workloads. However, the TA-GT pair and RF-GT pair only showed significant differences (*p* < 0.05) while workload was increased from 0 to 100 W.

**Table 3:** **Mean co-activation of four muscle pairs, in terms of crank angles (deg)**

Workload (Watts) →	0	50	100
**RF - BF**	24 (38) *,#	143 (51) *,◊	214 (61) #,◊
**TA - GT**	43 (69) #	72 (86)	130 (109) #
**RF - TA**	45 (45) *,#	98 (60) *,◊	174 (65) #,◊
**RF - GT**	47 (44) #	91 (60)	148 (75) #

Values are mean (±SD).

*: Statistical significance between workloads 0 and 50 W (p-value < 0.05).

#: Statistical significance between workloads 0 and 100 W (p-value < 0.05).

◊: Statistical significance between workloads 50 and 100 W (p-value < 0.05).

### Kinematics

To quantify the extent of change in movement kinematics across different workloads, two variables were considered: Mean Joint Angles (MA) and Ranges of Motion (ROM). MA was calculated as the average of joint angles across 20 consecutive full cycles of the pedal. Similarly, ROM was computed by averaging the difference between maximum and minimum angle values across 20 consecutive full cycles of the pedal. All variables were calculated for the flexion-extension of the hip (*Ө*_*H*_) and knee (*Ө*_*K*_), abduction-adduction of the thigh (*Ө*_*S*_), and dorsiflexion-plantarflexion of the ankle (*Ө*_*A*_). No statistically significant difference was observed while workload increased in any of these variables.

## Discussion

Muscle and joint coordination during upright cycling is well documented, experimentally [19–21] and theoretically in simulation studies [22–24]. In a theoretical study, Raasch et al. [24] developed a framework by simulating upright cycling and dividing muscles into four corresponding phase-controlled functional groups (PCFG), using the percentage of the total integrated EMG occurring in each phase. Researchers developed this framework to explain observed changes in EMG magnitude and timing during upright cycling in different conditions and, ultimately, to identify differences in the functional roles of muscles [7, 24]. In a more recent study, the PCFG framework was utilized by Hakansson and Hull [5] to study and compare muscle activity patterns and their functional roles at different pedaling rates during upright and semi-reclined cycling. The PCFG framework and its functional regions were adapted to fit the semi-reclined cycling position and to compare upright and semi-reclined cycling techniques [5]. There is, however, paucity in experimental research related to coordination and activation of muscles during semi-reclined cycling.

The purpose of this study was to evaluate the functioning and contribution of four lower limb muscles (i.e., RF, BF, TA, and GT) during constant cadence semi-reclined cycling performance across three different workload levels: 0, 50, and 100 W. Although no significant changes were observed in terms of movement kinematics, the EMG activity of the lower extremity was influenced by the increase in workload. These included the increase in the RF, BF, and TA durations of activity, as well as their EMG peak magnitude values. Although differences in the duration of activity and peak magnitude values in GT were not statistically significant, numerical values were greater at 100 W, compared to 0 W. Previous research [19] has also shown that changes in the workload affect the EMG burst onset and offset during cycling revolution. Baum and his colleague evaluated the effect of workload on lower limb muscles during upright cycling and observed an increased earlier onset in GT, RF, BF, TA, while GT, RF, BF showed earlier offset as workload increased. The results of our study are in agreement with Baum et al. [19] in that higher workload may potentially have a greater effect on these muscles when cycling. Our study showed that the overall patterns of muscle activity during cycling could change with changing workload, indicating that there may be different outcomes in measures such as strength (force generating capacity of a muscle) when cycling at different workloads. This proportional increase in the duration of activity and peak magnitude level with workload during cycling may further indicate additional recruitment of muscle fibers in order to maintain the constant cadence of pedaling at 60 rpm. This finding is consistent to a previous study by Trumbower and Faghri [2].

Our results indicated that increasing workload did not appear to have an effect on the EMG burst peak-timing for all four muscles during cycling. However, by increasing cadence, Baum and Li [19] demonstrated significant changes in the EMG peak-timing of BF, TA, and GT at a constant workload of 250 W. The results of our study are consistent with their findings.

Increase in workload, during semi-reclined cycling, significantly increased the level of co-activation between RF and BF muscles. In addition, co-activations of the upper and lower leg muscles were observed to be affected by an increase in workload. RF and TA showed greater levels of co-activation as workload increased. Studies have shown that during complex dynamic movements, such as cycling, running, and walking, the uniarticular muscles are primarily the power producers, whereas the biarticular muscles act to transfer power between the two joints. In addition, various conclusions have been reported regarding the activation of biarticular muscles (GT, BF, RF) during cycling [24–26]. Although this study did not examine the power production, nor its transfer, in lower limb muscles, our results show that biarticular GT appears to activate before TDC and remains active throughout the down stroke in all workloads (Figure 5). This observation is in agreement with the findings of Raasch et al. [24] who reported that GT is transferring energy from the limb to the crank in the same phase of the cycle. As previously reported, BF contributes in generating as well as transferring energy during the downstroke, which is the duration of activity for BF observed in our findings [24]. On the other hand, the principal function of RF is to generate energy during the upstroke while the energy generated to the limb is transferred to the crank by TA [24]. This pattern can explain the higher co-activation between the RF and TA muscle groups in higher workloads. Jorge and Hull [27] also reported a synergistic activity between RF and its antagonist, BF. Interestingly, the RF muscle, which acts as both the knee extensor and a hip flexor, remains active for a larger portion of the crank revolution. This information is important, since individuals with disabilities and the elderly may exhibit excessive co-activation of agonist and antagonist muscles during these complex activities and may compromise their effectiveness in transferring power.

From the results of this research and previous studies, it could be postulated that the different body orientation in semi-reclined cycling, compared to upright cycling, may result in different functional roles of each muscle group during pedaling. This is especially important since the design of a Functional Electrical Stimulation (FES)-induced cycle ergometer, which routinely is utilized for exercise by people with upper motor neuron lesions (e.g., spinal cord injury, stroke, etc.), is based on a semi-reclined cycle platform. Previous research has shown that while this unique system is helping some individuals with spinal cord injury (SCI), not all patients could fully benefit from this exercise. Trumbower and Faghri [2] demonstrated that muscle activation patterns in this semi-reclined cycling are different from upright cycling. They evaluated the timing and patterns of EMG activities of healthy individuals during semi-reclined cycling at different workloads. They further compared their findings to muscle activity patterns of upright cycling as well as commercially available FES-induced cycling systems and reported major differences in the timing and duration of lower limb muscle activities [2, 28]. These finding could have major implications on the efficacy of the commercially available FES-induced cycling systems. The FES activations of paralyzed muscles in these systems are based on the muscle activations during upright cycling, which may lead to cycling inefficiency reported in previous research [2, 28].

Consistent with previous research, our findings exhibited that various levels of workload do not affect the kinematic patterns during stationary semi-reclined cycling [3]. This may be due to the constraint nature of the stationary semi-reclined cycling; seating position was adjusted for every participant, based on his anthropometric characteristics. Additionally, participants were asked to keep their trunk straight during all sessions of the experiment. Consequently, alterations in kinematics were minimized and put all participants in consistently similar positions throughout the study.

## Conclusions

Cycling is an enjoyable aerobic activity that may provide cardiovascular fitness while reducing the stress on the joints, which often proliferates with aging or misuse. Individuals with compromised musculoskeletal system (e.g., decreased muscle strength, range of motion, and fitness), may potentially benefit from this types of exercise. However, consideration needs to be given to the types of cycling, level of workload, and cadence to reduce the potential side effects. Therefore, evaluation and understating of the cycling biomechanics and muscles’ activations and synchronizations during cycling could improve our knowledge in developing appropriate exercise regimen for at risk populations. This understanding is even more crucial for those with compromised joint condition. For example, following knee surgery the recommendation is to minimize transverse and varus-valgus force around the knee. Increases in workload may cause unsolicited co-contractions during cycling which could be too stressful on the joint and may further damage the tendons and soft tissues.

This study exhibited the effects of workload on the timing and duration of lower limb muscle activities during semi-reclined cycling; it also provided a better understanding of the relative contribution of these muscles in cycling performance. Future studies should be conducted to provide further insight into the muscular differences between the healthy and diseased populations during semi-reclined cycling and to provide proper modifications in the design of said populations’ exercise routines.

## Authors’ original submitted files for images

Below are the links to the authors’ original submitted files for images. Authors’ original file for figure 1 Authors’ original file for figure 2 Authors’ original file for figure 3 Authors’ original file for figure 4 Authors’ original file for figure 5 Authors’ original file for figure 6

## Abbreviations

BDC: Bottom-dead-center
BMI: Body mass index
EEA: Electromyography ensemble average
EMG: Electromyography
FES: Functional electrical stimulation
GMM: Generalized muscle moments
IPAQ: International physical activity questionnaire
IRB: Institutional review board
MA: Mean angles
RF: Rectus Femoris
BF: Biceps Femoris
TA: Tibialis Anterior
GT: Gastrocnemius Medialis
PAR-Q: Physical activity readiness questionnaire
PCFG: Phase-controlled functional groups
ROM: Ranges of motion
RPM: Rotations per minute
SCI: Spinal cord injury
SENIAM: Surface electromyography for the non-invasive assessment of muscles
TDC: Top-dead-center
W: Watts.
