# Supplementary figures and images for: Lower-extremity joint kinematics and muscle activations during semi-reclined cycling at different workloads in healthy individuals
Source: J Neuroeng Rehabil. 2014 Oct 17;11:146. doi: 10.1186/1743-0003-11-146 (PMC4216842; doi:10.1186/1743-0003-11-146)

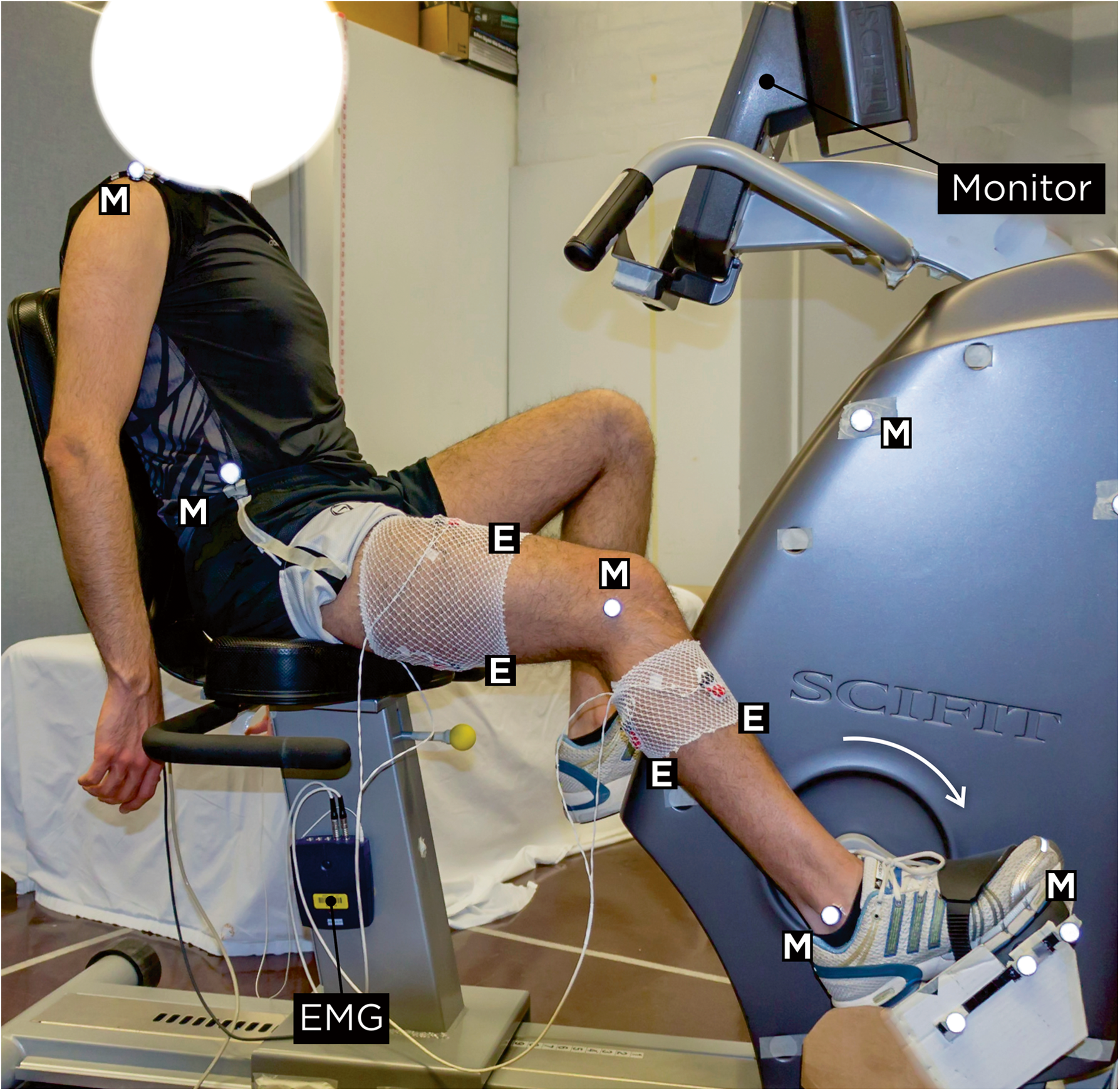

Supplement: Supplementary file 1 — Authors’ original file for figure 1 [file 12984_2014_665_MOESM1_ESM.tif]

Shoulder

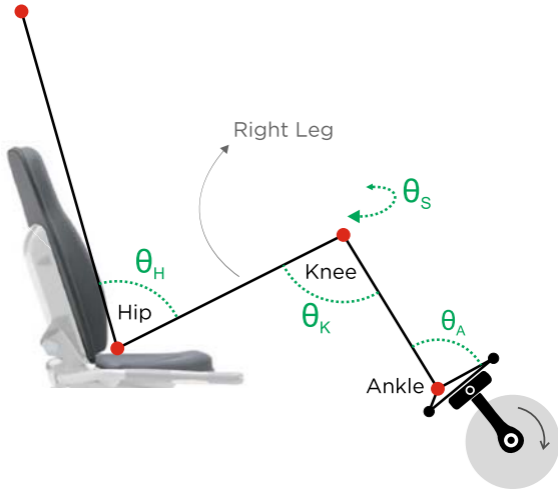

Supplement: Supplementary file 2 — Authors’ original file for figure 2 [file 12984_2014_665_MOESM2_ESM.pdf]

Top Dead Center (**TDC**)

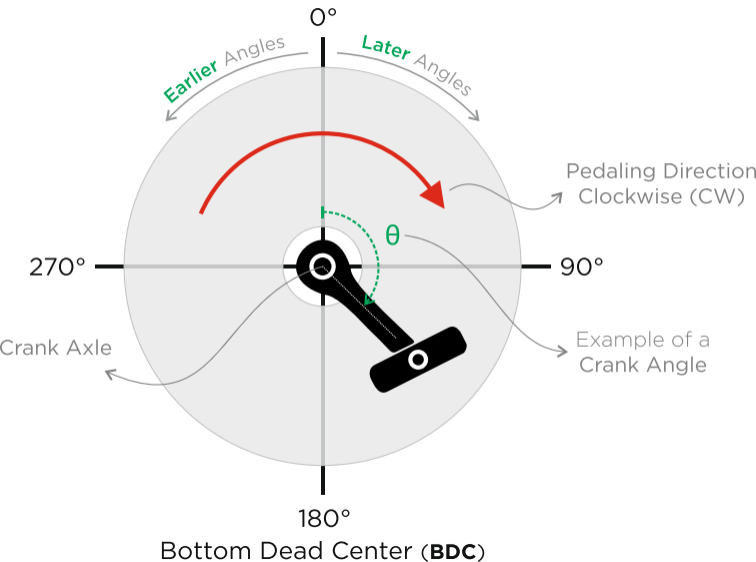

Supplement: Supplementary file 3 — Authors’ original file for figure 3 [file 12984_2014_665_MOESM3_ESM.pdf]

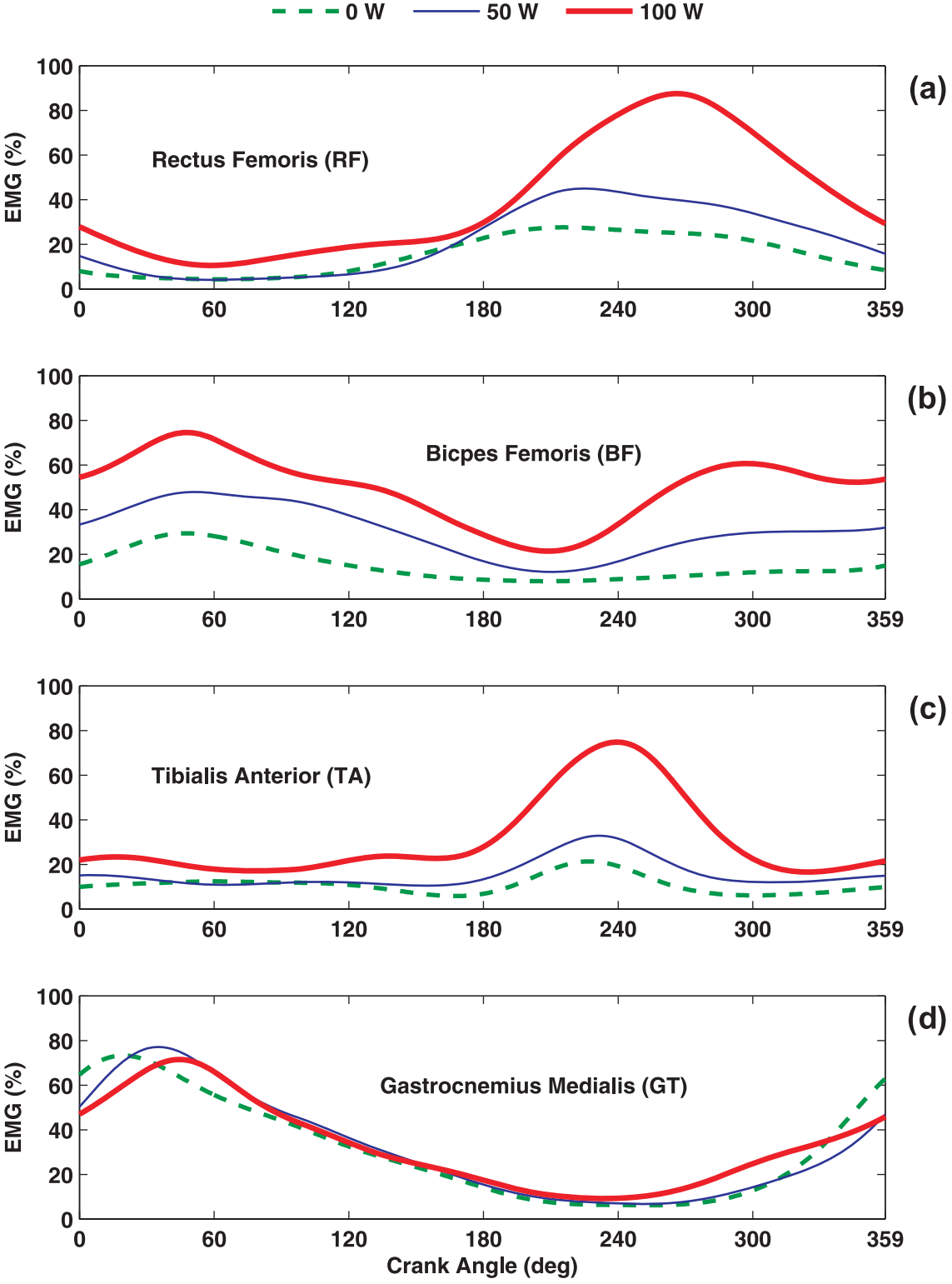

Supplement: Supplementary file 4 — Authors’ original file for figure 4 [file 12984_2014_665_MOESM4_ESM.pdf]

- 1 Rectus Femoris
- 2 Biceps Femoris
- 3 Tibialis Anterior
- 4 Gastrocnemius M.

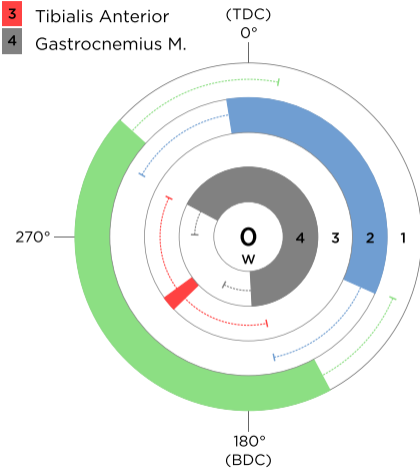

(a)

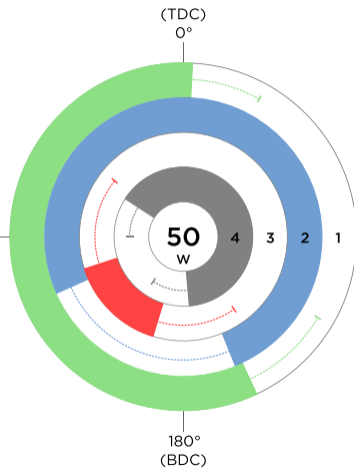

(b)

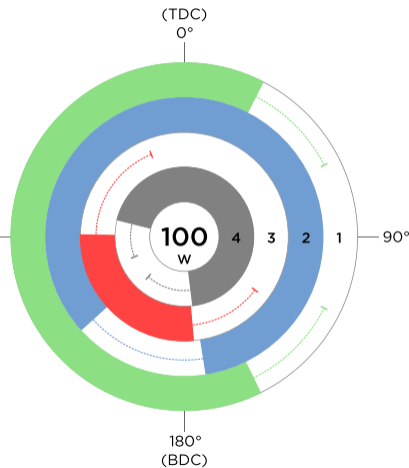

(c)

Supplement: Supplementary file 5 — Authors’ original file for figure 5 [file 12984_2014_665_MOESM5_ESM.pdf]

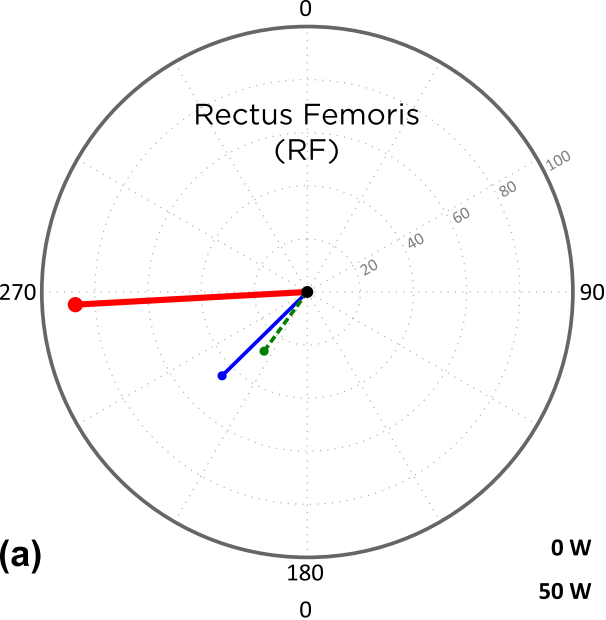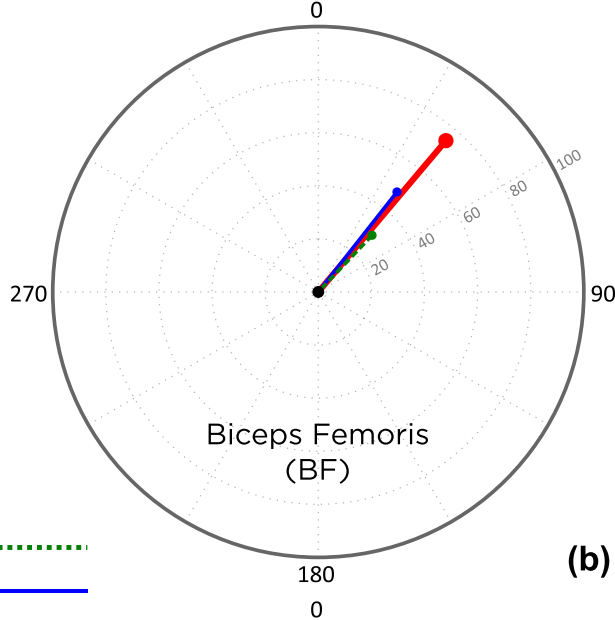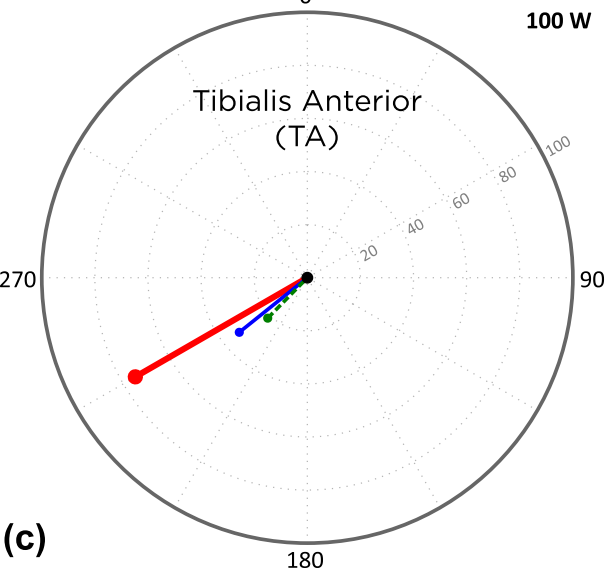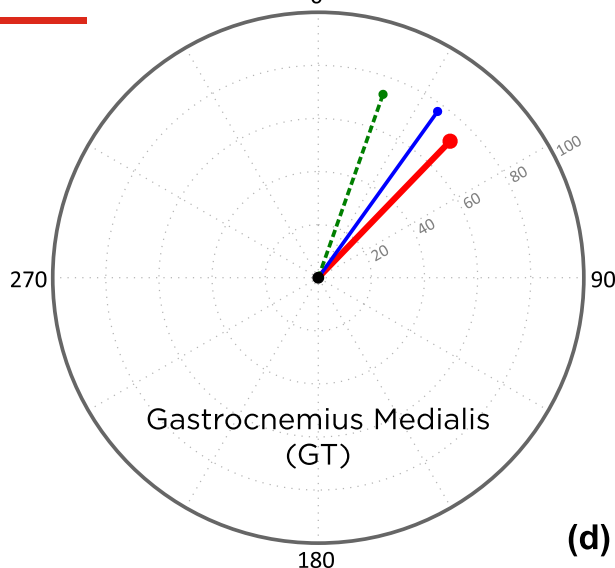

Supplement: Supplementary file 6 — Authors’ original file for figure 6 [file 12984_2014_665_MOESM6_ESM.pdf]
